# A Confirmatory Factor Analysis of the Brief Sense of Community Scale for Spanish-Speaking Older Adults in Puerto Rico

**DOI:** 10.1093/geroni/igaa057.356

**Published:** 2020-12-16

**Authors:** Tommy Buckley, Kyeongmo Kim, Denise Burnette

**Affiliations:** Virginia Commonwealth University, Richmond, Virginia, United States

## Abstract

Psychological sense of community is a concept used to describe how individuals feel about their community. The Brief Sense of Community Scale (BSCS) is an 8-item scale that includes these four domains: membership, needs fulfillment, emotional connection, and influence. It has been used in various contexts and was validated with young adults in Puerto Rico. The purpose of this study was to validate the BSCS for use with Spanish-speaking older adults in Puerto Rico. We conducted face-to-face interviews with a non-probability sample of 154 community- dwelling adults aged 60+ in Puerto Rico. BSCS is comprised of a 5-point likert-type scale with score values ranging from 0 (strongly agree) to 4 (strongly disagree) (total score range 0-32, mean= 24.75, SD= 6.04), and it showed good reliability in our sample (a=.85) and acceptable subscale reliability (membership, a=.85; needs fulfillment, a=.85; influence, a=.66; and emotional connection, a=.69). Five competing factor structures were tested based on prior research using confirmatory factor analysis (CFA). The CFA indicated that a four factor structure from the original scale was the best fit (χ² (16) =25.9; p=.06; RMSEA=.06; CFI=.98; TLI=.97; SRMR=.04). The BSCS showed significant correlations in the expected direction with quality of life (r=.41), social isolation (r=.34), loneliness (r=.27) and self-rated health (r=.17). We conclude that the BSCS is a valid and reliable scale for measuring psychological sense of community with community-dwelling Spanish-speaking older adults in Puerto Rico. Future research should confirm and extend our findings with other Spanish-speaking older adult populations.

